# Applying knowledge translation tools to inform policy: the case of mental health in Lebanon

**DOI:** 10.1186/s12961-015-0018-7

**Published:** 2015-06-06

**Authors:** Farah Yehia, Fadi El Jardali

**Affiliations:** Department of Psychiatry, American University of Beirut Medical Center, PO Box 11-0236, Riad El-Solh, 1107 2020 Beirut Lebanon; Department of Health Management and Policy, Faculty of Health Sciences American University of Beirut, PO Box 11-0236, Riad El-Solh, 1107 2020 Beirut Lebanon; Knowledge to Policy (K2P) Center, American University of Beirut, PO Box 11-0236, Riad El-Solh, 1107 2020 Beirut Lebanon; Center for Systematic Reviews of Health Policy and Systems Research (SPARK), American University of Beirut, PO Box 11-0236, Riad El-Solh, 1107 2020 Beirut Lebanon; Department of Clinical Epidemiology & Biostatistics, McMaster University, Hamilton, Ontario L8S 4L8 Canada

**Keywords:** Evidence-informed policymaking, Knowledge translation, Mental health, Mental illness, Primary healthcare

## Abstract

**Background:**

Many reform efforts in health systems fall short because the use of research evidence to inform policy remains scarce. In Lebanon, one in four adults suffers from a mental illness, yet access to mental healthcare services in primary healthcare (PHC) settings is limited. Using an “integrated” knowledge framework to link research to action, this study examines the process of influencing the mental health agenda in Lebanon through the application of Knowledge Translation (KT) tools and the use of a KT Platform (KTP) as an intermediary between researchers and policymakers.

**Methods:**

This study employed the following KT tools: 1) development of a policy brief to address the lack of access to mental health services in PHC centres, 2) semi-structured interviews with 10 policymakers and key informants, 3) convening of a national policy dialogue, 4) evaluation of the policy brief and dialogue, and 5) a post-dialogue survey.

**Results:**

Findings from the key informant interviews and a comprehensive synthesis of evidence were used to develop a policy brief which defined the problem and presented three elements of a policy approach to address it. This policy brief was circulated to 24 participants prior to the dialogue to inform the discussion. The policy dialogue validated the evidence synthesized in the brief, whereby integrating mental health into PHC services was the element most supported by evidence as well as participants. The post-dialogue survey showed that, in the following 6 months, several implementation steps were taken by stakeholders, including establishing national taskforce, training PHC staff, and updating the national essential drug list to include psychiatric medications. Relationships among policymakers, researchers, and stakeholders were strengthened as they conducted their own workshops and meetings after the dialogue to further discuss implementation, and their awareness about and demand for KT tools increased.

**Conclusions:**

This case study showed that the use of KT tools in Lebanon to help generate evidence-informed programs is promising. This experience provided insights into the most helpful features of the tools. The role of the KTP in engaging stakeholders, particularly policymakers, prior to the dialogue and linking them with researchers was vital in securing their support for the KT process and uptake of the research evidence.

**Electronic supplementary material:**

The online version of this article (doi:10.1186/s12961-015-0018-7) contains supplementary material, which is available to authorized users.

## Background

Health systems worldwide often fail to use evidence optimally in their policymaking processes, creating a gap between knowledge and actual practice [1]. Recently, there has been an increased interest in ensuring health policymaking – especially in low- and middle-income countries (LMICs) that have many health challenges but limited resources – is guided by the best available research evidence to improve public health [2, 3].

Knowledge translation (KT) has emerged as a means for closing the gap between knowledge and practice. Straus et al. [1] formally defined KT in 2009 as a “dynamic and iterative process that includes the synthesis, dissemination, exchange, and ethically sound application of knowledge to improve health, provide more effective health services and products, and strengthen the healthcare system”.

As a result, knowledge translation platforms (KTPs) have been established in several LMICs [2]. KTPs form partnerships among policymakers, researchers, civil society groups, and other stakeholders and focus their efforts on two distinctive and complementary activities: preparing policy briefs and convening policy dialogues [3]. In response to the gaps in evidence-informed health policymaking in Lebanon, a KTP, named the Knowledge to Policy Center (K2P), was established in 2013 at the American University of Beirut. It draws on a breadth of synthesized evidence and context-specific knowledge about a priority topic to impact policy agendas and action. K2P does not restrict itself to research evidence but integrates multiple types of knowledge to inform policy dialogue, including grey literature and expertise of stakeholders.

A policy brief brings together global research evidence (from systematic reviews) and context-specific knowledge to inform deliberations about health policies and programs [4]. Policy briefs address multiple barriers that hinder the uptake of research evidence by policymakers in LMICs, such as the common perception that the available evidence is not relevant or user-friendly. Policy briefs package research evidence and contextualize it for policymakers according to the local health system [3]. Policy dialogues use policy briefs as primary input, which allows for the best available research evidence to be considered, along with the tacit knowledge and perspectives of the key health actors who may be affected by the decisions to be taken [5]. Policy dialogues facilitate interaction among policymakers, researchers and key stakeholders, and such interactions are known to promote the use of evidence in policymaking [3].

Nevertheless, it has not yet been determined how design and content features affect the usefulness of these KT tools [3]. Only a few studies exist on the effectiveness of KTP activities in strengthening evidence-informed health policymaking in LMICs, and their findings are inconsistent [3].

**Fig. 1:**
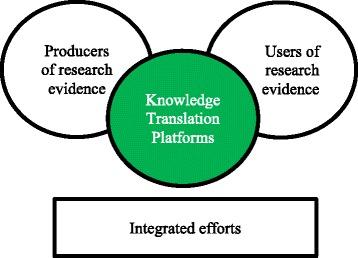
Integrated model for linking research into action

Lavis et al. [6] propose an “integrated” KT model (Fig. 1) in which the KTP fosters linkage and exchange efforts across a health system. In this model, the KTP works to align the research efforts of researchers with the needs of policymakers; it infuses public dialogue with an understanding of research evidence. In this study, the K2P Center played the role of a KTP: an intermediary between researchers on the one hand and the Ministry of Public Health of Lebanon (MOPH) and other key stakeholders on the other. They collaborated to tackle the issue of mental health in primary healthcare (PHC) settings in Lebanon.

This study seeks to gain a better understanding of the influence of KT tools and aims to inform initiatives towards promoting evidence-informed policymaking by taking mental health as a case study. It examines the process of influencing the mental health agenda in Lebanon through the application of KT tools and the use of a KTP as a first-of-its-kind initiative in Lebanon and the region.

### The case of mental health in Lebanon

Mental disorders are a leading direct cause of disease burden worldwide [7]. In Lebanon, one in every four adults suffers from at least one mental disorder throughout their lives [8]. Only a minority of those obtain treatment, and there are prolonged delays to seeking treatment, ranging from 6 years for mood disorders to 28 years for anxiety disorders [8]. Such delays are critical because early assessment and intervention can positively alter the natural progression of mental disorders into chronic and disabling conditions. The burden of mental disorders extends to other diseases as they worsen the outcome of co-occurring conditions such as cancer, heart disease, and diabetes [9]. In addition, mental disorders are costly to national economies in terms of expenditure and loss of productivity [10]. Further, when present in caretakers, mental disorders impact children’s health and development [10–12]. In Lebanon, the exposure to war-related trauma, internal conflicts, and political instability have increased the prevalence of several mental disorders [13–15].

The private sector provides most of the mental health services in Lebanon, and the MOPH contracts with private hospitals to pay for needy patients who require inpatient care. Three mental hospitals and five psychiatric units within general hospitals exist. The essential list of psychotropic medications includes antipsychotics, anxiolytics, mood stabilizers, and antiepileptics, which are supplied by the MOPH to PHC centres for free. Lebanon does not have enough psychiatrists (1.5 psychiatrists per 100,000 population), with the majority of those working in private practices or for-profit institutes. Two-thirds of other psychosocial professionals (psychologists, other medical doctors, nurses, social workers) work in the public sector according to the WHO AIMS Report of 2010 [16]; financing mental health remains mainly an out-of-pocket expenditure [16–19].

Prior to 2014, a dedicated mental health program did not exist within the MOPH, despite the eagerness of the ministry to be more active in this area. Initial efforts at training non-specialized PHC staff first began in 2008 by the International Medical Corps but were discontinued due to several challenges [18], and in 2012 WHO restarted preparations for integration of the mental health Gap Action Programme (mhGAP) at the PHC level. Mental health legislation (Lebanese Act no. 72-9/9/1983 “Welfare Act and Protection and Treatment of Mentally Ill Patients”) was outdated, not implemented, and did not fully guarantee the rights of mentally ill patients to proper access to care [13, 17]. A draft law was prepared in 2008 to update Law 1983/72; however, it has been pending parliamentary approval ever since.

In September 2013, the K2P Center partnered with the MOPH and identified mental health as a top health policy priority through a priority-setting exercise that the K2P Center aims to conduct on a regular basis. In parallel, the MOPH had been preparing a National Mental Health Program, which was officially launched later in April 2014. The integration of mental health into PHC was one of the program’s key priorities.

The Kingdon model proposes that the public policy process includes multiple components flowing in different streams; problems, policies (solutions), and political will. When these streams intersect, a window of opportunity opens up and policymakers decide to act [20]. This is what happened in late 2013 in Lebanon with mental health, as the K2P Center had identified mental health as a top health policy priority, and the MOPH was preparing to establish the National Mental Health Program. Additionally, social pressure was growing by civil society groups who had launched a national mental health awareness campaign in October 2013. Therefore, the K2P Center, based on its criteria for priority-setting, identified a window of opportunity to influence policymaking and partnered with the MOPH to achieve it.

## Methods

**Fig. 2:**
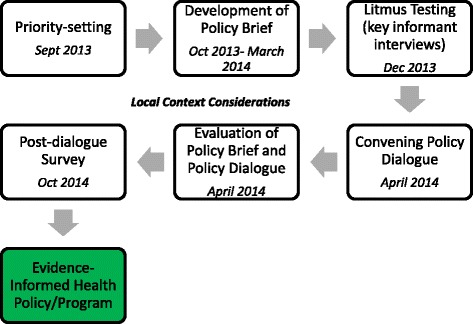
The process of using knowledge translation tools to inform mental health policy in Lebanon

This study employed the following KT tools: 1) priority-setting, 2) development of a policy brief to address the mental health problem, 3) semi-structured interviews with policymakers and key informants, 4) convening of a national policy dialogue, 5) evaluation of the policy brief and policy dialogue, and 6) post-dialogue survey (Fig. 2).

A K2P Core Team was formed and consisted of a health systems expert, a mental health expert, a policymaker, and a researcher. The K2P Core Team used a methodology for the application of KT that was based on the SURE Guides and SUPPORT Tools [5, 21]. SURE Guides are a comprehensive set of tools providing a step-by-step approach to support key points and decisions in developing policy briefs [21]. SUPPORT Tools assist those responsible for making decisions about health policies based on research evidence and those who support these decision makers [5].

### Priority-setting

One of the first activities conducted by the K2P Center when it was established in 2013 was a priority-setting exercise in partnership with the MOPH, in which mental health was identified as a top health policy priority. K2P has a set of adapted criteria for selecting priority topics, and mental health satisfied the majority of those criteria, including the topic is important, there is public interest on it, there is sufficient local evidence to quantify the problem, there is relevant research evidence and available options to address it, there is an opportunity for change, it has national and regional relevance, and it has been recognized as a policy challenge.

### Development of policy brief

To harness published evidence as well as tacit knowledge, the K2P Core Team developed a search strategy that was validated by an expert librarian. The first step in the search strategy was a literature review aiming at identifying the specific problems and its underlying factors. This was done by seeking information about the mental healthcare system arrangements (in terms of governance, financing, and service delivery), indicators of the burden of mental illnesses, and relevant legislations and developments in the field of mental health in Lebanon in recent years. A search limited to documents published from the year 1996 to 2014 was conducted in MEDLINE, The Cochrane Library, and Google Scholar to retrieve relevant articles. The following keywords were used: mental health, mental health services, psychiatry-y/ic, psychology-y/ic, mental disorders, mental illness, Middle East, and Lebanon. WHO reports, including the WHO Mental Health Atlas [22], were also reviewed, in addition to the grey literature such as the websites and reports of the Lebanese MOPH. A documentation review of the relevant legislation, draft laws, and the National Mental Health Strategy was also conducted.

The second step in the search strategy was performed to gather information on the different elements of the policy approach to address the problem. A search for relevant systematic reviews was conducted per element using the following databases: Health Systems Evidence, Cochrane Database of Systematic Reviews, The Cochrane Library, and SUPPORT. When systematic reviews were not available, single studies were retrieved from MEDLINE. The quality of systematic reviews was assessed using the AMSTAR scale, and the journal rankings for single studies were determined according to ISI Web of Knowledge. The details of the search strategies are presented in an additional file (Additional file 1).

A draft outline for the policy brief was prepared, between October and December 2013, based on the evidence synthesis, stating the identified problem and the three elements of the policy approach to address it. The brief was reviewed using the K2P Litmus Test (detailed below).

The policy brief was then prepared by synthesizing the best available evidence about the problem and viable solutions through the comprehensive literature review. The gathered evidence was contextualized according to the health system of Lebanon using input gathered from context experts and policymakers throughout the process of drafting the brief. The policy brief was then used to inform deliberations of a national policy dialogue. It was circulated to dialogue participants prior to the dialogue to serve as the starting point for off-the-record deliberations.

### Semi-structured interviews with policymakers and key informants

A K2P Litmus Test, adapted from the McMaster Health Forum [21], was performed through a semi-structured interview with a purposive sample of key informants in December 2013. The Litmus Test is somewhat similar to the method used by the McMaster Health Forum in that it is a key informant interview approach which allows the researchers to 1) gather input about the draft outline for the policy brief to support a stakeholder dialogue, 2) help frame the problem, and 3) identify other key informants who might be able to provide further input. The test was translated into Arabic and its questions were contextualized according to the topic at hand (in this case, the issue of mental health in PHC settings).

The sampling frame included stakeholders in mental health from different disciplines: policymakers, researchers in mental health and public health, healthcare providers including mental health specialists, professional associations, civil society organizations, PHC representatives, and public and private insurers. A total of 12 informants were identified and targeted; out of these, 10 agreed to participate (Table 1). Findings from the interviews were summarized using a thematic analysis approach and were then used as input to review the policy brief outline.

**Table 1:** Description of stakeholders participating in Litmus Test and Policy Dialogue

Stakeholder category	Number who were consulted in Litmus Testing	Number who attended Policy Dialogue
Policymakers or civil servants (Ministry of Public Health)	2	2
Civil society organizations and patient advocacy group	2	4
Primary healthcare representatives	1	3
Researchers in public health and mental health	2	4
Healthcare providers including mental health specialists	2	6
Representatives of professional associations	1	2
Health insurer	0	1
International health organizations	0	2
Total	10	24

### Convening of national policy dialogue

A policy dialogue, pre-circulated by the policy brief, was convened on April 24, 2014 under the title “Securing access to quality mental health services in primary healthcare in Lebanon” [23].

The K2P Core Team identified a group of 24 stakeholders to participate in the dialogue based on the following criteria: 1) they would bring to the dialogue unique views and experience to bear on the issue and learn from the research evidence and from others’ views and experience, and 2) after the dialogue they would champion within their respective institutions the actions that would address the challenge creatively. The dialogue ensured a fair representation among policymakers, civil society organizations, PHC representatives, researchers, healthcare providers including mental health specialists, and representatives of the National Social Security Fund (insurer) and International Health Organizations (Table 1).

The dialogue followed the Chatham House rule to allow for frank off-the-record discussions [5]. A facilitator from the K2P Core Team assisted with the deliberations. The dialogue aimed to foster discussion informed by the best available evidence, rather than to achieve consensus. Observations of these deliberations were recorded by a member of the K2P Core Team. Participants were asked to complete an evaluation survey at the end of the dialogue. Two weeks following the dialogue, a summary was disseminated to participants to help them in their follow-up actions.

### Evaluation of policy brief and policy dialogue

The policy brief evaluation survey was adapted from Lavis et al. [4]. It was circulated to participants once via email a few days prior to the dialogue and once again at the dialogue for those who did not complete it via email. The survey consisted of 10 items rating how helpful different aspects of the brief were, as well as how well the brief achieved its purpose of presenting the available research evidence to inform the policy dialogue, using a scale from 1 to 7 (1 being very unhelpful, 4 being neutral, and 7 being very helpful).

The policy dialogue evaluation survey was also adapted from Lavis et al. [5]. It was circulated to participants at the end of the dialogue. The survey consisted of 8 items rating how helpful they found different aspects of the dialogue, as well as how well the dialogue achieved its purpose of supporting a full discussion of relevant considerations about the problems and its policy approach in order to inform action, on a scale from 1 to 7 as in the policy brief evaluation survey. Responses from the surveys were descriptively analyzed.

### Post-dialogue survey

Six months following the dialogue, a short survey was circulated to six key participants purposively selected from different agencies that championed the issue of mental health in the country, each in their own capacity. They were therefore deemed by the K2P Core Team as the most knowledgeable about the latest developments. The survey aimed to follow-up on the deliberations that took place, track progress, and identify implementation issues pertaining to the integration of mental health into PHC.

## Results

The below results describe findings based on the synthesized evidence in the policy brief, followed by findings from the discussions that took place at the policy dialogue. All resulting documents (policy brief, policy dialogue summary, and others) were produced in both English and Arabic.

### Framing of the problem and its underlying factors

The policy brief defined the overall problem as the limited access of a large proportion of individuals suffering from mental health problems and their families to mental healthcare services in PHC settings in Lebanon. The current health system arrangements do not ensure equitable access to high quality mental health services.

From the perspective of service delivery, the only way to access mental healthcare in the public sector is through psychiatric institutions [16], and this does not promote prevention or community integration. The integration of mental health into the PHC network is still weak [17, 18]. Though essential psychotropic medications are supposed to be distributed to PHC centres for free, in reality they are often unavailable there. Centres also lack assessment and treatment protocols necessary to provide frontline basic psychological and psychiatric interventions, and only a few of them regularly refer patients to specialized mental healthcare clinics when needed. In addition, evidence-based multidisciplinary treatment practices for mental health patients are rarely implemented.

From a financing perspective, coverage of mental health services in Lebanon is extremely low as the primary source of financing for mental health is out-of-pocket payments. There are disparities in the coverage of public funding mechanisms for mental health services, and private insurance and mutual funds explicitly exclude these services from their policies [19]. The dependence of the current system on out-of-pocket expenditure for mental health creates an access barrier for individuals with low incomes and who are at highest risk [11].

During the policy dialogue, participants confirmed the underlying factors forwarded in the policy brief. Deliberations around the problem definition, which was presented in the policy brief, consumed the largest amount of time. Participants agreed that there is a problem in the lack of trained healthcare professionals at PHC centres who can recognize mental health conditions and provide basic mental health services. However, a number of participants argued that the issue is not only the limited supply of services but also the limited demand for services, which is related to people’s poor awareness or knowledge about their mental health conditions.

Participants reframed the problem as the limited knowledge of people suffering from mental health problems and their families about mental illness, as well as their limited access to mental healthcare services in PHC settings in Lebanon.

### Elements of a policy approach to address the problem

Three elements of a comprehensive approach were presented in the policy brief; we refer to them as “elements” of a comprehensive approach rather than “options” because they are not mutually exclusive. This term was adapted from the McMaster Health Forum.

*Element 1*: *Integrate mental health into PHC service provision by developing an essential health services package to be a guaranteed minimum*.

WHO strongly recommends the integration of mental healthcare into general PHC services as the most viable way of ensuring that people have access to the mental healthcare they need [24]. Integration of mental health into PHC can be implemented through collaborative care, which aims to develop closer working relationships between PHC and specialist healthcare providers. There are different ways and models through which collaborative care can be implemented, including task-shifting, case management, and liaison psychiatry [25–31]. Compelling evidence has demonstrated the effectiveness and cost-effectiveness of PHC-led service systems for the treatment of mental disorders [24].

Deliberation about element 1 during the policy dialogue revealed that participants agreed with the need for a minimum service package. On choosing between the different models of collaborative care (task-shifting, case management, liaison psychiatry), participants noted that one model, such as task-shifting, is promising but would not be enough and that a combination of different models will most probably be needed in the context of Lebanon. PHC providers learn the general principles of care and the key actions such as establishing communication and building trust, conducting assessments, management of cases, referrals and follow ups for priority conditions. Participants recommended scaling up of the package for the most prevalent mental health conditions in Lebanon and using guidelines and protocols to standardize mental health practice in PHC settings. According to some participants, integrating screening as part of a horizontal program may induce over-diagnosing and thus is a potential harm of element 1.

It is important to note that integration does not reduce psychiatrists’ work, but rather changes the nature of their work to focus on more complex and severe psychiatric cases, while less complex cases can be managed by trained non-specialist health workers. This is what the evidence in the policy brief [25, 28] showed and was acknowledged by some participants at the dialogue.

*Element 2*: *Expand coverage of mental health services in the PHC setting*, *as well as coverage for specialist services for patients referred by PHC centres*, *through reimbursement by third party payers according to a capitation payment system*

Under capitation, payment is made based on the number of patients to whom care is provided. Only one high-quality systematic review addressed payment mechanisms for mental health integrated into PHC, but it concluded that there is not yet sufficient evidence to determine which reimbursement system leads to better health outcomes or cost-effectiveness [27]. Although no other systematic reviews were identified specifically about reimbursement systems for mental health in PHC, our search did retrieve other systematic reviews on reimbursement mechanisms for PHC in general.

During the deliberation about element 2, participants noted that the MOPH is already considering some form of capitation as a new model of financing PHC services in general, and not only mental health services. Issues raised by participants included the following: the need to take the patient’s perspective into consideration regarding capitation and the need for non-financial incentives for providers. Such incentives could include research opportunities for academicians, the privilege of being part of a national and international initiative, or in a material form such as the MOPH helping providers secure their Continuing Medical Education through the Order of Physicians. Another issue raised was the need to engage community stakeholders in the catchment area of the PHC centre, who could themselves participate in the funding of the centre’s mental health services.

*Element 3*: *Recognize parity between mental health and physical health by developing and implementing appropriate legislation*, *including issuing the draft law for the licensing of psychologists*, *as well as the proposed Mental Health Act for the protection of psychiatric patients*’ *rights*

Although no systematic reviews were identified about policies related to integration of mental health into PHC, the search identified compelling evidence from numerous single studies on enacting mental health legislation. One of the most important benefits identified by several high-quality studies was that enacting national mental health-related policies resulted in a decline in suicide and self-harm rates.

During the deliberations about this element, participants discussed the status of the Mental Health Act at the time of the dialogue. The political situation had been impeding the progress of the draft law, as it was still pending due to the idleness of the parliament and shortage of quorum. The Act was yet to be reviewed by the parliamentary committee and it focused on the following areas: protection of the patient, regulating the work of the psychiatrist, involuntary hospital admissions, freedom of the patient to select the kind of treatment within the hospital and to get a second medical opinion, the role of the government in treatment and follow-up after discharge, and setting procedures for the use of electroconvulsive therapy and physical restraints. Participants recognized that there might be an opportunity to lobby for this Act through patient advocacy initiatives.

### Policy brief and policy dialogue evaluation results

Nine out of 24 participants completed the policy brief evaluation survey, despite repeated attempts to administer the survey. The low response rate can be attributed to the participants’ busy schedules and time limitations. Average scores on all items were positive, ranging between 6 and 7. The results of the evaluation of the policy dialogue also yielded favourable results, whereby 9 out of 24 participants responded to the survey and average scores on all items were high, ranging also between 6 and 7. Results on the evaluation of the policy brief and policy dialogue are presented in Additional file 2.

The specific aspects of the policy dialogue that were found most helpful by participants were the graded-entry format that the brief employed (a list of key messages, executive summary, and a full report), the fact that the brief described different features of the problem and three elements of an approach for addressing it, and that the brief described what is known, based on synthesized research evidence, about each of the three elements and what the gaps were. Participants also found helpful that the brief described key implementation considerations and took quality considerations as well as local applicability considerations into account when discussing the research evidence.

As for the beneficial aspects of the policy dialogue, participants found most helpful that the dialogue was informed by a pre-circulated policy brief, and that it engaged a facilitator to assist with the deliberations. Other aspects deemed helpful were the fact that the dialogue brought together many parties who could be involved in or affected by future decisions related to the issue, and that it allowed for frank and off-the-record deliberations.

### Post-dialogue survey results

Six key stakeholders were purposively selected from different agencies deemed as the most knowledgeable about the latest developments in the field of mental health in Lebanon, and four of them responded to the post-dialogue survey. The stakeholders represented the following agencies: WHO, MOPH, PHC, and mental healthcare providers and patient advocacy groups. The results of the survey revealed key actions undertaken by stakeholders following the dialogue. A mental health and psychosocial support taskforce was established to coordinate efforts across Lebanon. A WHO mhGAP adaptation workshop was held in June 2014 and, accordingly, an adaptation and translation of the WHO mhGAP training manual to fit the Lebanese context were conducted. In the same month, a meeting with the directors of PHC centres was held to secure their buy-in. This meeting was followed by initiating the mhGAP training-of-trainers and training in 45 out of 198 centres across Lebanon, along with the initiation of the Psychological First Aid training. In addition, the national drug list which includes mental health medications has been reviewed and updated, and performance indicators of the project were identified and integrated into the Ministry’s Health Information System.

In parallel to those developments, other actions contributed to strengthening mental health in Lebanon. In July 2014, the MOPH secured a 20 million euro grant from the European Union to strengthen PHC services, including mental health, which will support the integration of mental health services into PHC. The national mental health strategy draft was reviewed. Two public debates of the draft mental health law were held in the summer of 2014 and modifications to the draft law were made based on evidence-informed practices and stakeholder input. The first national awareness campaign for suicide prevention was launched in September 2014. In addition, a national survey of Lebanese adults’ knowledge and attitudes towards mental illness was conducted between October and December 2014.

Respondents also highlighted challenges they encountered in translating the elements that were discussed at the dialogue into action. This validated some of the challenges that were predicted in the policy brief based on previous international experiences with integration efforts. As cited in the policy brief, stakeholders faced opposition from psychiatrists to the integration of mental health services in PHC. In order to address this challenge, the roles of non-specialized staff and referral pathways were thoroughly discussed and clearly defined during the mhGAP adaptation workshop. Another expected barrier cited in the policy brief was the fear of specialists that non-specialized staff would not be sufficiently competent, and this was indeed faced in Lebanon. Accordingly, non-specialized staff members are being thoroughly trained under regular supervision for at least a year to make sure they are competent in assessing, diagnosing and managing some cases at PHC level, and referring cases that need specialized care. Finally, the policy brief cited opposition from PHC management as a barrier to integration efforts in many countries. To overcome this potential challenge, PHC managers were engaged at an early stage, i.e., before the trainings.

## Discussion

The application of KT tools to tackle the problem of mental health and help generate evidence-informed policies and programs was a promising experience and the first of its kind in Lebanon and the region. This experience served as a demonstration of the KT process to Lebanese policymakers and proved to be a valuable means for bringing evidence into their hands.

In this case study of mental health in Lebanon, a window of opportunity for influencing policymaking was identified. Within the priorities of its newly established National Mental Health Program, the MOPH had outlined what needs to be done, and the K2P Center played the role of providing the means through which to put the program into action; in other words, the how.

The policy brief and policy dialogue helped inform policymaking at the government level. The policy dialogue, informed by a pre-circulated policy brief, has helped trigger or support multiple actions by stakeholders directly related to the integration of mental health into PHC, as well as actions aimed at strengthening other aspects of mental health in Lebanon (such as legislation and public awareness). Participants exhibited great momentum for mobilizing efforts to address the issue of mental health in Lebanon, as evident by the numerous steps taken in the 6 months that followed the dialogue. Participants also pointed out that the policy dialogue was an important opportunity for a large and diverse group to deliberate about the problem and its elements. A similar finding was reported in a study published by Moat et al. [3] in which participants favoured the fact that policy dialogues bring together many individuals who could be involved in, or affected by, future decisions related to the problem at hand and options for addressing it.

This case study has shown that the use of KT tools strengthened the relationships among policymakers, researchers and stakeholders; different stakeholders continued the discussion that began at the policy dialogue through follow-up meetings and workshops that they conducted by themselves to delve further into implementation issues. The use of KT tools also increased their awareness about the importance of evidence-informed policymaking initiatives. This was translated into an increased demand for KT products by policymakers (i.e., “user pull” efforts), and these findings are consistent with the outcomes reported by a study on 10 KTPs in different LMICs [2]. After the dialogue, the K2P Center was approached by the MOPH for consultation on other public health priority topics, such as the issues of Syrian refugees’ health and food safety in Lebanon.

An important observation was that the framing of the problem proved to be critical. The key informant interviews and the policy dialogue helped in refining the framing of the problem. The policy dialogue also served to validate the evidence synthesized in the policy brief, whereby element 1 of the policy approach for addressing the problem was the most supported by evidence as well as dialogue participants. Here, it is interesting to point out that the participants’ choice of the element to pursue might not have been influenced only by the strength of the evidence supporting it, but also by the feasibility of its application in the context of Lebanon. Specifically, participants may have steered away from element 3, for example, because political factors in Lebanon were identified as a barrier that had been hindering the enactment of mental health legislation for years. The policy dialogue was instrumental in contextualizing the elements. This further emphasizes the importance of taking local context into consideration when discussing evidence and deciding on options to pursue. Previous studies have shown that the uptake of evidence by policymakers and its usefulness in supporting evidence-informed health policies is influenced by contextual factors, such as the institutions, interests, and values in the local context [2, 32].

Follow-up after the dialogue, which in this case study was done through disseminating a policy dialogue summary and conducting a post-dialogue survey, is extremely important. In fact, related studies have reported that follow-up activities can help continue building the capacity of stakeholders to address the policy issue. Such activities include disseminating dialogue summaries, providing customized debriefing to specific stakeholders on the implications of the dialogue, or offering a year-long service of providing newly published evidence including systematic reviews [5, 33].

There are several lessons learned from this KT experience. One lesson was that working within an “integrated” knowledge model was absolutely essential, whereby stakeholders were engaged from the very beginning and the KTP helped establish a partnership between researchers and policymakers. It was observed by the researchers that engaging key stakeholders early on through the K2P Litmus Test played an important role in securing their buy-in; such politics were just as important and time-consuming as writing the policy brief itself. This finding is consistent with the experience of other KTPs in LMICs [2], indicating that strong leadership support, especially from governmental policymakers, and their willingness to participate in KT initiatives was key to achieving outcomes and bringing about change [2].

This experience helped provide insight on the most helpful aspects of the policy brief and policy dialogue. A study on the use of KT tools in six different LMICs found that every key feature of the evidence briefs and deliberative dialogues was viewed favourably by most respondents, regardless of the countries in which they were used or the issues they addressed [3]. This was validated in this mental health case study whereby all the features of the policy brief and policy dialogue were viewed positively by participants, according to the results of the evaluation surveys.

One of the most helpful features of the policy dialogue reported by participants was that it engaged a skilled facilitator to assist with the deliberations. Similar findings have been reported in a study on KTPs in LMICs, which revealed that skilled human resources were a key component that helped KTPs engage in deliberative processes [2]. Another recent study noted that the most important design feature to retain for future dialogues was having a skilled facilitator as an unbiased agent [34].

As positive as this experience of using KT tools for supporting evidence-informed policymaking has been, the K2P Core Team has observed that these tools might not be enough on their own to push health policy agenda in Lebanon. Taking mental health as an example, it was clear that for such a topic to be properly tackled, attitudes and perceptions need to be addressed not only at the policymakers’ level but also within the general public. Stakeholders at the policy dialogues stressed the importance of raising public awareness about mental health to complement the value added by KT tools. To arrive at healthcare system arrangements that guarantee proper provision of psychiatric and psychological services, the stigma of seeking professional mental healthcare needs to be tackled as well as people’s recognition of the signs and symptoms of common mental disorders.

### Limitations

A limitation worth stating is the low response rate to the policy brief and dialogue evaluations, whereby only nine out of 24 participants responded to the questionnaires, despite repeated attempts to administer the survey. This can be attributed to the participants’ busy schedules and to the time constraints at the dialogue. Therefore, it is advisable to allocate around 10 minutes of a policy dialogue’s agenda specifically for participants to complete the evaluation surveys. Nevertheless, six participants provided positive feedback about the KT experience in a 3-min videotaped interview immediately after the dialogue.

It is also important to caution that this study does not claim any cause–effect relationship stemming from the KTP process. Other factors may have contributed to the positive changes that followed, such as the clear recognition and interest of the MOPH in promoting mental health and the efforts made in previous years by different researchers and civil society groups to advance mental health in the country.

The 6-month period between the dialogue and the post-dialogue survey was appropriate for examining short term developments following the KTP activities, but a longer period might be needed to capture further changes that might have materialized. An additional limitation is the absence of service users or their representatives from the Litmus Test stakeholder groups; this was remedied by including a patient advocacy group at the dialogue. Nevertheless, it may be worthy, in future KTP initiatives, to consult patients through focus groups designed specifically for patients allowing them to voice their true opinions and concerns.

## Conclusions

This study has demonstrated how the use of KT tools in Lebanon played a key role in helping mental health move forward on the policy agenda. Mental illness poses a significant burden on the Lebanese community, and yet, access to mental health services in PHC settings remains inadequate and inequitable. This problem was highlighted and a policy approach was presented and discussed by a multidisciplinary group of stakeholders. Moving forward, more work needs to be done on measuring not only the outcomes, but the impact of implementing evidence-informed policies using KT (e.g., better mental health in the Lebanese community, better access to care for people living with mental disorders). Integrating monitoring and evaluation components of KT from the start allows us to measure whether and how research evidence was used in the policy, and whether the policy achieved its intended outcome and health impact. Strengthening the research base on the impacts of KT in improving health outcomes would solidify the argument for promoting evidence-informed health policies.

## Additional files

Additional file 1:
**Search strategy.**
Additional file 2:
**Policy dialogue and policy brief evaluation results.**

## Abbreviations

K2P: Knowledge to Policy Center
KT: Knowledge translation
KTPs: Knowledge translation platforms
LMICs: Low- and middle-income countries
mhGAP: Mental health Gap Action Programme
MOPH: Ministry of Public Health of Lebanon
PHC: Primary healthcare
