# Supplementary material for: Applying knowledge translation tools to inform policy: the case of mental health in Lebanon
Source: Health Res Policy Syst. 2015 Jun 6;13:29. doi: 10.1186/s12961-015-0018-7 (PMC4461900; doi:10.1186/s12961-015-0018-7)
Supplement: Additional file 1: — Search strategy. [file 12961_2015_18_MOESM1_ESM.docx]

**Search Strategy**

For the first element, three searches were conducted whereby the following keywords were used:

In Health Systems Evidence:

• Mental health + primary care

• Mental health + primary care + integration of services

• Mental health + primary healthcare + integration of services

• Mental health + Task shifting

• Mental health + Task shifting + primary care

• Psychiatry + primary care

• Psychology + primary care

In Cochrane Database of Systematic Review, browsed by topic:

• Mental health

• Public health > health related systems

• Effective practice/health systems

In Cochrane Library, with “economic evaluation” limit:

• Integration of mental health into primary care

• “task shifting” AND “mental health”

• “non specialist” AND “mental health”

• “non specialist” AND “psychiatric”

For the second element, three searches were conducted whereby the following keywords were used:

In Health Systems Evidence:

• Mental health (with topic “financial arrangement” selected)

• Mental health + primary care + reimbursement

• Mental health + primary care + reimbursement mechanism

• Mental health + primary care + payment

• Mental health + primary care + payment model

• Psychiatry/ist + primary care + payment

• Psychology/ist + primary care + payment

• Task shifting + reimbursement

• Task shifting + payment

• Task shifting + remunerating providers

• Mental health + capitation

• Reimbursement + collaborative care

• Payment + collaborative care

• Mental health + coverage

• Mental health + insurance

• Specialists + financial arrangements

In Cochrane Database of Systematic Reviews, browsed by topic:

• Effective practice/health systems> financial arrangements

In SUPPORT:

• Database of SUPPORT summaries > Health systems

As for the third element, four searches were conducted whereby the following keywords were used:

In Health Systems Evidence:

• Mental health (with topic “governance arrangement” selected)

In Cochrane Database of Systematic Reviews, browsed by topic:

• Effective practice/health systems> governance arrangements

• Mental health + policy

• Mental health + governance

In SUPPORT:

• Database of SUPPORT summaries > Health systems

In MEDLINE:

Database: Ovid MEDLINE(R) without Revisions <1996 to February Week 2 2014>

Search Strategy:

1 Mental Health/lj [Legislation & Jurisprudence] (51)

2 Mental Disorders/ (57392)

3 Health Policy/ (34528)

4 Community Mental Health Services/ec, lj, og [Economics, Legislation & Jurisprudence, Organization & Administration] (2943)

5 "Delivery of Health Care, Integrated"/ec, lj, og [Economics, Legislation & Jurisprudence, Organization & Administration] (4740)

6 Mental Health/ (13688)

7 5 and 6 (23)

8 Mental Health Services/ec, lj, og [Economics, Legislation & Jurisprudence, Organization & Administration] (7797)

9 Primary Health Care/ec, lj, og [Economics, Legislation & Jurisprudence, Organization & Administration] (10451)

10 8 and 9 (375)

11 4 or 7 or 10 (3329)

12 3 and 11 (183)

13 limit 12 to (english and (classical article or comparative study or evaluation studies or government publications or journal article or legal cases or legislation or meta analysis or multicenter study or observational study or randomized controlled trial or systematic reviews or technical report) and last 14 years) (128)

14 1 or 2 (57426)

15 3 and 14 (733)

16 limit 15 to (english and (classical article or comparative study or evaluation studies or government publications or journal article or legal cases or legislation or multicenter study or observational study or randomized controlled trial or systematic reviews or technical report or validation studies) and last 14 years) (509)

17 from 13 keep 2,10,14-15,17,23-24,26,28-29,40,43,50,61-63,65,71,74,78,83-89,98-99,114,128 (31).
