# Supplementary material for: Applying knowledge translation tools to inform policy: the case of mental health in Lebanon
Source: Health Res Policy Syst. 2015 Jun 6;13:29. doi: 10.1186/s12961-015-0018-7 (PMC4461900; doi:10.1186/s12961-015-0018-7)
Supplement: Additional file 2: — Policy dialogue and policy brief evaluation results. [file 12961_2015_18_MOESM2_ESM.docx]

**Policy Dialogue and Policy Brief Evaluation Results**

**Policy Dialogue Evaluation Results**

| Very unhelpful | Moderately Unhelpful | Slightly unhelpful | Neutral | Slightly helpful | Moderately helpful | Very helpful |
| --- | --- | --- | --- | --- | --- | --- |
| 1 | 2 | 3 | 4 | 5 | 6 | 7 |

9 out of 24 participants answered the dialogue evaluation questionnaire.

| **Questions** | **Average score** |
| --- | --- |
| 1. The policy dialogue was informed by a pre-circulated policy brief. | **7** |
| 2. The policy dialogue was informed by discussion about the full range of factors that can inform how to approach a problem, possible elements of an approach for addressing it, and key implementation considerations. | **6** |
| 3. The policy dialogue brought together many parties who could be involved in or affected by future decisions related to the issue. | **6** |
| 4. The policy dialogue aimed for fair representation among policymakers, stakeholders, and researchers. | **6** |
| 5. The policy dialogue engaged a facilitator to assist with the deliberations. | **7** |
| 6. The policy dialogue allowed for frank, off-the-record deliberations by following the Chatham House rule: “Participants are free to use the information received during the meeting, but neither the identity nor the affiliation of the speaker(s), nor that of any other participant, may be revealed.” | **6** |
| 7. The policy dialogue did not aim for consensus. | **6** |
| 8. The purpose of the policy dialogue was to support a full discussion of relevant considerations (including research evidence) about a high-priority policy issue in order to inform action. How well did the policy dialogue achieve its purpose? | **6** |
| 9. I have been working in my current position for _____ years. | **8 (years)** |

| **Broad role category** | **Specific role category** | **Number of respondents** |
| --- | --- | --- |
| Policymaker | Public policymaker (i.e., elected official, political staff, or civil servant) in the national government |  |
|  | Manager in a district/region (if it does not have independent policymaking authority) |  |
|  | Manager in a healthcare institution (e.g., hospital) |  |
|  | Manager in a non-governmental organization (NGO) | 3 |
| Stakeholder | Staff/member of a civil society group/community-based NGO | 1 |
|  | Staff/member of a health professional association or group | 2 |
|  | Staff of a donor agency |  |
|  | Representative of another stakeholder group |  |
| Researcher | Researcher in a national research institution | 1 |
|  | Researcher in a university | 3 |
|  | Researcher in another institution |  |
| Other |  | 1 |

**Policy Brief Evaluation Results**

9 out of 24 answered the brief evaluation questionnaire.

| Questions | **Average scores** |
| --- | --- |
| 1. The policy brief described the context for the issue being addressed. | **6** |
| 2. The policy brief described different features of the problem, including (where possible) how it affects particular groups. | **7** |
| 3. The policy brief described three elements of an approach for addressing the problem. | **7** |
| 4. The policy brief described what is known, based on synthesized research evidence, about each of the three elements and where there are gaps in what is known. | **7** |
| 5. The policy brief described key implementation considerations. | **6** |
| 6. The policy brief took quality considerations into account when discussing the research evidence. | **6** |
| 7. The policy brief took local applicability considerations into account when discussing the research evidence. | **6** |
| 8. The policy brief did not conclude with particular recommendations. | **6** |
| 9. The policy brief employed a graded-entry format (e.g., a list of key messages and a full report). | **7** |
| 10. The purpose of the policy brief was to present the available research evidence on a high-priority policy issue in order to inform a policy dialogue where research evidence would be just one input to the discussion. How well did the policy brief achieve its purpose? | **7** |
| 11. I have been working in my current position for _____ years. | **7 (years)** |

| **Broad role category** | **Specific role category** | **Number of respondents** |
| --- | --- | --- |
| Policymaker | Public policymaker (i.e., elected official, political staff, or civil servant) in the national government |  |
|  | Manager in a district/region (if it does not have independent policymaking authority) |  |
|  | Manager in a healthcare institution (e.g., hospital) | 1 |
|  | Manager in a non-governmental organization (NGO) | 3 |
| Stakeholder | Staff/member of a civil society group/community-based NGO |  |
|  | Staff/member of a health professional association or group | 3 |
|  | Staff of a donor agency |  |
|  | Representative of another stakeholder group |  |
| Researcher | Researcher in a national research institution | 2 |
|  | Researcher in a university | 2 |
|  | Researcher in another institution | 1 |
| Other |  | 1 |

**Open-ended comments:**

- provide a glossary of the key terms
